# Visual Neuropsychology in Development: Anatomo-Functional Brain Mechanisms of Action/Perception Binding in Health and Disease

**DOI:** 10.3389/fnhum.2021.689912

**Published:** 2021-05-31

**Authors:** Silvio Ionta

**Affiliations:** Sensory-Motor Lab (SeMoLa), Department of Ophthalmology-University of Lausanne, Jules Gonin Eye Hospital-Fondation Asile des Aveugles, Lausanne, Switzerland

**Keywords:** vision, movement, brain, child, sensorimotor

## Abstract

Vision is the main entrance for environmental input to the human brain. Even if vision is our most used sensory modality, its importance is not limited to environmental exploration. Rather it has strong links to motor competences, further extending to cognitive and social aspects of human life. These multifaceted relationships are particularly important in developmental age and become dramatically evident in presence of complex deficits originating from visual aberrancies. The present review summarizes the available neuropsychological evidence on the development of visual competences, with a particular focus on the associated visuo-motor integration skills in health and disease. With the aim of supporting future research and interventional settings, the goal of the present review is to constitute a solid base to help the translation of neuropsychological hypotheses into straightforward empirical investigations and rehabilitation/training protocols. This approach will further increase the impact, ameliorate the acceptance, and ease the use and implementation of lab-derived intervention protocols in real-life situations.

## Neural Correlates of Vision

**Figure 1:**
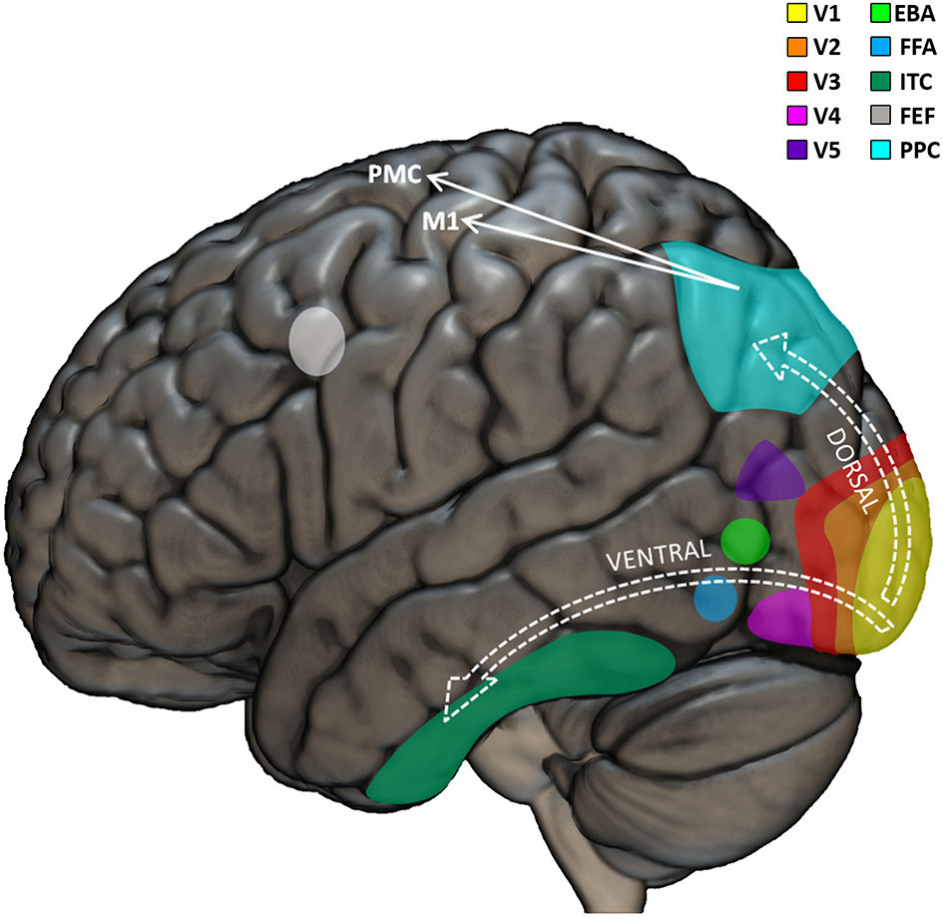
Visual neuropsychological model. Graphical representation of the main cortical regions involved in visual perception and visuo-motor coordination. The visual input is first processed by the primary visual cortex (V1). Further processing is performed by the extrastriate visual regions (V2, V3, V4, and V5) which triggers the recruitment of the dorsal or ventral stream as a function of whether or not the visual input needs to be used to perceive or move in the environment, respectively. EBA, extrastriate body area; FFA, fusiform face area; ITC, inferior temporal cortex; FEF, frontal eye field; PPC, posterior parietal cortex; M1, primary motor cortex; PMC, premotor cortex.

Visual perception permeates our life, not only for merely gathering information about the environment, but also for having important influence on our motor skills. Revealing the neural mechanisms of the multifaceted relationships between vision and other domains of human life, visual neuropsychology goes beyond the traditional consideration of vision as a passive function, and rather highlights how visual competences can impact typical and atypical development at a more systemic, dynamic, and integrated level. The neurobiological machinery that brings from light to vision starts in the eyes, where the photoreceptors of the retina are able to selectively respond to the photons of light (entered through the cornea and projected to the retina) and “translate” them into neural signals. These signals are transported by the optic nerves to subcortical structures (the lateral geniculate and pulvinar nuclei of the thalamus) which relay signals mainly to the visual cortex, in the posterior part of the brain, but also to the superior colliculus in the midbrain (Shipp, 2004). The occipital lobe is further organized in several sub-regional areas, including the striate primary visual cortex (V1) and a series of interconnected, extra-striate, and progressively more specialized areas for higher-level processing of visual input (Figure 1). Thus, while V1 is sensitive to basic features of the visual input, such as line orientation, motion direction, and depth perception, the secondary visual cortex (V2) receives fibers from V1, projects to the third visual cortex (V3), and is already able to perform figure/background distinctions (Qiu and Von Der Heydt, 2005; Maruko et al., 2008), to process illusory contours (Von Der Heydt et al., 1984; Anzai et al., 2007), and to build binocular disparity (Von Der Heydt et al., 2000). V3 projects to areas out of the occipital lobe, including the posterior parietal cortex (Stepniewska et al., 2016) and the inferior temporal cortex (Ponce et al., 2017), and is sensitive to global motion (Braddick et al., 2001), covering larger portions of the visual field with respect to V1 (Lui et al., 2006). The fourth visual cortex (V4) it tightly connected to V1 and V2 (Liu et al., 2020) and projects mainly to the inferior temporal cortex (Bohon et al., 2016). It is involved in color perception, object recognition, and is sensitive to top-down attentional modulation (Roe et al., 2012). The fifth visual cortex (V5) receives input from V1, V2, V3, as well as from the thalamus (Ungerleider and Desimone, 1986; Felleman and Van Essen, 1991; Sincich et al., 2004; Warner et al., 2010) and projects to the superior temporal gyrus (Handa et al., 2017; Handa and Mikami, 2018), the frontal eye fields (Machner et al., 2010) and lateral intraparietal cortex (De Azevedo Neto and Amaro Junior, 2018). Some fibers reach V5 directly from the thalamus, bypassing V1 (Warner et al., 2012). Encoding speed and direction of visual input (Dubner and Zeki, 1971; Maunsell and Van Essen, 1983), V5 is mostly important for motion perception and smooth guidance of eye movements (Dursteler et al., 1987) as well for “building” a continuous perception of moving targets and scenes instead of a “crystallized” vision of distinct frames (Hess et al., 1989; Baker et al., 1991). The sixth visual cortex (V6) is located medially and connected to parietal and pre/post-central regions (Shipp et al., 1998; Galletti et al., 2001; Luppino et al., 2005; Smith et al., 2018; Serra et al., 2019) of the brain is responsible for “subtracting out” the visual input related to self-motion from the rest of the visual perception (Pitzalis et al., 2013), as well as for visually guiding movements (Pitzalis et al., 2015).

Traditional neuropsychological models of visual perception indicate that the several interconnections among the visual regions of the brain can be broadly classified according to two functionally different main streams: the well-known ventral and dorsal streams (Tong, 2003). The “what” *ventral* stream would pass signals from V1, V2, V3, V4, up to the inferior temporal cortex and would be implied mainly in object recognition Conversely, the “where” *dorsal* stream would comprise connections between V1, V2, V3, superior/medial temporal sulcus, and parietal cortex and would be particularly important for neurally encoding the visuo-spatial and motion-related aspects of visual input (Hickok and Poeppel, 2004; Almeida et al., 2010; De Haan and Cowey, 2011; Goodale, 2013). Lesions in the ventral stream produce recognition deficits such as prosopagnosia (the impossibility to recognize faces) (Mayer and Rossion, 2007). Lesions in the dorsal stream determine visuo-motor deficits, such as optic ataxia (impaired visuo-motor coordination, e.g., impossibility to reach objects despite preserved visual and motor skills separately) (Himmelbach et al., 2009). This sharp dichotomy between ventral and dorsal streams has been progressively smoothened (Rossetti et al., 2017), including the identification of bidirectional interactions between the streams (Greulich et al., 2020), especially in the context of adaptive behavior (Goodale et al., 2005) and visuo-motor skills (Van Polanen and Davare, 2015). Interestingly, different visuo-motor sub-pathways have been identified in the dorsal stream: the dorso-dorsal stream would be recruited for online action control; the ventro-dorsal stream would have be involved in higher-level cognitive functions including action understanding (Rizzolatti and Matelli, 2003). Altogether, it seems clear that the fine precision of our visual skills and their importance in action-related mechanisms are reflected in the high complexity of the involved neural architecture.

## Neuro-Behavioral Developments of Visual Skills

Taking into consideration the temporal aspects of the development of the visual streams, it appears that the pace at which the ventral and the dorsal streams grow would be different already in pre-born age, indicating that the ventral stream would mature more quickly than the dorsal stream (Tadros et al., 2015). Indeed, already at birth the ability of newborns to notice that a visual event occurs, even if the classification of “what” that even is still need further cortical maturation, has been considered an example of the importance of subcortico-cortical visual functions (Bronson, 1974). In addition, the fact that newborns are particularly attracted by face-like visual stimuli (Simion et al., 2011) and, especially, by familiar faces (Bushneil et al., 1989), suggests the functioning of thalamo-V5 connections which would bypass V1. Then, during the first 6 months, more specific functions associated with neural activity in V1 progressively emerge in an ordered sequence. The first functions are sensitivity to orientation, followed by the ability to perceive directional motion, and finally binocular interactions, e.g., for depth perception (Braddick and Atkinson, 2011).

### Basic Functions

#### Visual Acuity

Visual acuity refers to our ability to perceive fine visual details. To reach adult levels, visual acuity rapidly develops during the first months of life and keeps improving up to 3 or 4 years (Norcia and Tyler, 1985; Banks and Dannemiller, 1987). Together with visual acuity, at birth also contrast sensitivity (the ability to discriminate light and dark) is below adult levels and progressively improves during the first months (Banks and Salapatek, 1978). From an ecological perspective, newborns don't need to perceive small details of far objects, but rather they need to recognize persons relevant for them (e.g., parents). Thus, newborns' relatively low visual acuity anyways allows them to efficiently interact with the environment relevant at their scale, even if their visual performance would be rated as “blindness” according to adult scales. Their low visual acuity might derive from the immaturity of foveal photoreceptors, retina, and eye-brain pathways, which quickly develop in early life, including denser concentration and better sensitivity of photoreceptors (Yuodelis and Hendrickson, 1986) and larger neural sprouting between the eyes and the brain (Braddick and Atkinson, 2011).

#### Vernier Acuity

The simple visual acuity provides the means to perceive small details, but it does not take into account the spatial relationship between such details. This capacity is defined “vernier acuity” and refers to the ability to perceive spatial incongruence, e.g., misalignment, with a resolution even higher than simple visual acuity. The behavioral aspects of vernier acuity have been long known, since the seminal work by Ewald Hering at the end of the nineteenth century (Strasburger et al., 2018). As it goes beyond the physical features of the eye, the vernier acuity is one of the examples of the importance of cortical dynamics in supporting visual skills (Manny, 1988; Skoczenski and Norcia, 1999). Indeed it progressively improves faster than the simple visual acuity (Zanker et al., 1992; Brown, 1997), in parallel with the maturation of the cortico-subcortical networks responsible for the integration of spatial relationships between different objects and their parts, and it reaches maturity much later than simple acuity (Skoczenski and Norcia, 2002). Interestingly, Braille reading triggers a progressive improvement of vernier acuity on tactile perception (Loomis, 1979), suggesting the influence of neuroplastic changes driven by specific habits.

#### Accommodation

Both visual acuity and vernier acuity depend on accommodation, the possibility to adapt the focus to the distance of the object by contracting or relaxing the muscles of the eye lens. Accommodation at birth is limited to objects located within a range of 40–50 cm (Horwood and Riddell, 2008), with a possibly parallel ongoing attentional limitation (Downey et al., 2017). At the neural level, accommodation is associated with brain activity in the visual cortex (Mirzajani et al., 2017) and relies on an extended cortico-cerebellar network, including links of the visual cortex with cerebellar hemispheres/vermis and temporal cortex (Richter et al., 2000), as well as with precentral and frontal regions (Lv et al., 2020).

#### Color

All visual functions would not reflect the real world if they would not comprise information about colors. Color perception is strongly based on the early activity of cone receptors in the eyes from the first months of life (Brown, 1990). It has roots in the development of cortical functions starting from V2 and V3 (Ting Siok et al., 2009) to allow the proper use of color information including, for example, high-level functions such as emotion (Yoto et al., 2007) or aesthetics (Maglione et al., 2017).

### Integrative Functions

#### Contours and Motion

One the most important information about the environment refers to the contours of an object: where an object ends and another one or the background start. This type of information is not present in the raw visual input, but it is rather built by neural responses in V1 (Hubel and Wiesel, 1977). Newborns start to discriminate orientation and therefore contours within the first weeks (Slater et al., 1988), even before visual event related potentials (VERPs; the stereotyped brain response to a standardized basic visual input) associated with contours can be recorded from their brains, between 3 and 8 weeks from birth (Braddick et al., 1986). Considering the importance of sensitivity to contours in development (Candy et al., 2001), VERPs can be fundamental measures for early detection of at-risk populations (Atkinson et al., 2008). Another fundamental information to efficiently understand the environment is the ability to distinguish static and moving objects. Like contours, motion sensitivity is the result of a cortical construction based on the neural responses in the visual cortices. Usually, newborns start to perceive motion a bit earlier than 10 weeks (Braddick et al., 2003), when their VERPs start to be detectable (Wattam-Bell, 1991). The anatomical maturation necessary to discriminate contours and motion occurs in the first months of life, depending on neural sprouting and synaptic establishment (Huttenlocher et al., 1982). The functional development of sensitivity to orientation and motion (plus binocularity) occurs in sequential order and based on neurally distinct pathways. For example, even if newborns show some degrees of motion sensitivity in relatively early stages, this ability relies mostly on sub-cortical structures (Morrone et al., 1999).

#### Global Motion

The perception of motion alone does not account for the complexity of the environment, where different objects can move at a different pace and/or according to different spatio-temporal patterns. The capacity to perceive such a complexity seems to appear already between the first 2–6 months of life (Kellman and Spelke, 1983; Arterberry and Yonas, 2000; Johnson and Mason, 2002), with a specific ability for the recognition of human action-like motion, or biological motion (Booth et al., 2002). Such a relatively late development together with the need to convey information from larger areas of the visual field suggest that global motion skills rely on a neural architecture starting in V1 (Robertson et al., 2014) and further extending to a broader brain network (Koyama et al., 2005), likely encompassing V2 and V3 (Furlan and Smith, 2016), as well as V5 (Giaschi et al., 2007) and possibly also more frontal motor regions (Saygin et al., 2004; Wuerger et al., 2012). Already in about 5-year-old children, the perception of global motion is independent from simple visual acuity (Chakraborty et al., 2015) and is strictly related to visuo-motor skills (Chakraborty et al., 2017). In addition, sensitivity to global motion is neurally dissociable from global form perception (Vachon et al., 2009), possibly being linked to interregional neural connections (Pavlova et al., 2005). A direct way to assess global motion skills is the so-called “motion coherence sensitivity” test (Newsome and Pare, 1988), which evaluates the ability to recognize target motion patterns within a background of differently moving dots. During the first months of life, motion coherence sensitivity increases progressively (Mason et al., 2003), the associated VERPs show specificity for global motion (Wattam-Bell et al., 2010), and V5 is selectively activated by global motion in connection with other motion-related areas (Biagi et al., 2015).

#### Static Forms vs. Global Motion

There seem to be a clear segregation between sensitivity to global motion and static forms, as shown by the earlier readiness of VERPs associated to global motion than to static forms (Wattam-Bell et al., 2010), the larger variability of thresholds for global motion than for static forms (Braddick et al., 2016), and qualitative difference between global motion and static forms skills ranging up to adolescence (Meier and Giaschi, 2014). At the neural level, while sensitivity to global motion seems to recruit mainly the dorsal stream, the perception of static forms is mainly bound to the ventral stream. Accordingly, only the development of global motion perception (not static forms) correlates with anatomo-functional growth of neural connections between the parietal and frontal lobes, beyond the solely visual cortex (Braddick et al., 2016, 2017). This suggests that global motion perception is a higher-order function recruiting also sensorimotor integration mechanisms. Such a conclusion is further supported by the observation that the performance in global motion (not static forms) correlates with visuo-motor skills already in developmental age (Braddick et al., 2016) and that aberrancies in its parieto-frontal neural architecture might be at the origin of the so-called “dorsal stream vulnerability” in developmental deficits (Spencer et al., 2000).

#### 3D and Depth Perception

The ability to merge and coordinate information from the two perspectives of each eye provides one of the means to perceive three-dimensionality and is a peculiar cortical function, not happening in earlier levels of the visual input's processing. In children, binocularity emerges at about 3 months (Thorn et al., 1994), depending on ocular convergence (Downey et al., 2017), cortical maturation (Elberger and Smith, 1985), neural plasticity (Chalupa, 2004), specific neurotransmitters (Kameyama et al., 2010; Krahe and Medina, 2010), and cortico-cortical interactions, both in humans (Jurcoane et al., 2007) and other mammals (Dehmel and Lowel, 2014). The failure of one or more of these cortical mechanisms can contribute to the creation of the conditions for developing binocularity-related functional deficits, such as strabismus (Berman and Murphy, 1981; Freeman et al., 1982; Di Stefano and Gargini, 2002). Together with binocularity, depth perception relies on a number of visual abilities, including shape/shade segregation, sensitivity to differential texture density, interposition of near/far surfaces, all of which start to be present between 4 and 7 months (Yonas et al., 2002), and it keeps progressing in parallel with the development of fine visuo-motor skills both in health (Braddick and Atkinson, 2013) and disease (Grant et al., 2014). A specific function tightly linked to depth perception is the ability to identify an object with respect to its background. Such a figure-ground discrimination can be based, for example, on the sensitivity to different textures between the object and the background which starts to emerge in the first month of child's life (Brooks and Clair, 1971; Wattam-Bell, 1992), keeps evolving up to adultness (Anderson et al., 2016), and is sensitive to age-related ocular diseases like macular degeneration (Tran et al., 2011). A related effect refers to the perception of the so-called “illusory contours,” proper visual illusions inducing the illusory perception of edges without physical borders, as shown by the pioneering work by Gaetano Kanisza and his famous illusory triangle (Kanisza, 1955). Children start to be sensitive to the Kanisza triangle in the first 3–5 months of life (Kavšsek, 2002; Otsuka and Yamaguchi, 2003), with increasing sensitivity up to adolescence (Bondarenko et al., 2010), possibly in parallel with improved cortico-cortical interactions (Ffytche and Zeki, 1996), increased intracortical dynamics in V2 and in V2-V5 exchanges (Grossberg, 2014), and exchanges between the different compartments of the visual cortex (Weigelt, 2007). Altogether, bidirectional interactions seem to be in place between children's improvements in perceiving basic visual features and their developments in higher-level functions beyond the mere visual perception. Such interactions typically start in the first month of life (Granrud, 2006) and keep evolving up to about 10 years (Nardini et al., 2010; Dekker et al., 2015).

#### Face Perception

The human face is probably the most salient visual stimulus in our life. The evolutionary and adaptive importance of the recognizing, understanding, and interpreting human faces is demonstrated by the existence of a region in the extrastriate cortex of the human brain, specifically dedicated to processing face-related visual input: the fusiform face area or FFA (Kanwisher et al., 1997). Behavioral evidence shows that sensitivity to faces is one of the first visual abilities in newborns (Goren et al., 1975; Ferrari et al., 1986), is based on contrast polarity (Rosa Salva et al., 2012), can be functional even with a relatively low resolution at short distances (Von Hofsten et al., 2014), and can be shaped by life experience (Cobbett and Snelgrove-Clarke, 2016). At the neural level, the early appearance of the strong bias toward faces suggests that it is based on subcortical mechanisms aiding the newborn to fixate a face which in turn would favor a frequent exposure to faces and the associated development of selective cortical processing. This idea is supported by the supposed presence of visual pathways which would allow face perception by directly connecting the thalamus and the amygdala to FFA, bypassing V1 in both the human (Morris et al., 2001) and the primate brain (Bourne and Morrone, 2017). Despite cortical electrophysiology suggests that the FFA is sensitive to observation of faces already at 4 months (De Heering and Rossion, 2015), face-related neural response (De Haan et al., 2003) and cortical specialization (Peelen et al., 2009; Deen et al., 2017) seem less pronounced in children than adults.

#### Body Perception

Together with face perception, also the visual perception of the human body plays a crucial role in daily life. Similarly to the fusiform face area, and in obvious anatomical closeness, the extrastriate body area (EBA) is functionally-defined brain regions, specifically sensitive to the observation of the human body (Downing et al., 2001) and part of the lateral occipitotemporal cortex possibly overlapping with V5 (Ferri et al., 2013). The inhibition (Urgesi et al., 2007; Candidi et al., 2008) or lesion (Peelen and Downing, 2007) of EBA support its causal implication in selectively respond to the observation of human bodies. Not only is EBA important for body-related visual processing, but also it is involved in higher-level visual cognition related to the human body, including identity attribution (Myers and Sowden, 2008), emotional resonance (Ionta et al., 2020), and mental imagery (Arzy et al., 2006; Costantini et al., 2011; Perruchoud et al., 2016). Even if the development of EBA in the life span remains largely unexplored, recent evidence suggests that the development of the body-specific responsiveness of EBA can take several years. Indeed neuro-functional differences of EBA can be noticeable between 6 and 8 vs. 9–12 year-old children (Walbrin et al., 2020), and the development of EBA can be affected by neurological disorders in early age (Okamoto et al., 2017).

### Visuo-Motor Interactions

Moving is one of the most direct and evolutionary relevant reason to have vision. Thanks to movements we can preserve our body, and therefore our life, for example by escaping dangers and reaching targets. Even if these functions can be technically possible also without vision, in typical conditions human beings are historically hardwired to vision. Therefore, it is not surprising that a large part of visual functions is subsequently used to control movements, as suggested by tight visuo-motor resonance already in early age (Lepage and Théoret, 2007). The neural architecture for such visuo-motor couplings would be present already at birth (Meltzoff and Moore, 1977) and it would be promoted by the repeated exposure to sensorimotor events (Cook et al., 2014), being its development further depending on experience (Simpson et al., 2014).

#### Eye and Head Movements

The first visuo-motor interactions in the newborns comprise eye and head movements, followed by postural adjustments, manual exploration, and locomotion (Adolph and Franchak, 2017). Eye movements constitute a fundamental visuo-motor interaction, combining the perceived changes from the environment (vision) with a rudimental motor reaction (eye movements). The superior colliculus in the midbrain plays a central role in such rudimental visuo-motor interactions, initiating the saccades (Hainline et al., 1984) and being connected to cortico-subcortical circuits to disengage fixation during saccades (Braddick et al., 1992). The fixation disengagement reaches functional maturity between 2 and 5 months (Hood and Atkinson, 1993), possibly reflecting the maturation of frontal cortical regions (Csibra et al., 1998). In order to track moving objects, it is necessary to (i) stabilize the target image on the retina and (ii) follow its displacements. The retinal stabilization can be achieved thanks to the optokinetic nystagmus, which subsequently needs to be inhibited in order to smoothly pursue the target's movements. In newborns, noting such ability depends on the features of the target, with smooth pursuit movements exhibited even at a few weeks of life, but only with slow and large moving targets (Phillips et al., 1997). The fact that infants can also anticipate where a moving target will go by staring at the expected location, supports that rudimental cortical mechanisms for early visuo-motor interactions are already in place in early age (Rosander, 2007).

#### Eye-Hand Coordination

It is not a secret that the ability to grasp objects contributed fundamentally to render humans one of the most evolutionary successful species worldwide. Grasping is the result of complex interactions between sensory perceptions and motor control, the largest part being taken by the coordination between vision and hand movements. Such an eye-hand coordination widely permeates daily life, including object manipulation, environmental exploration, and social interaction. Without tight eye-hand links, it is doubtful that fundamental human activities like writing or driving (often given for granted, but in fact not obvious), would have evolved at such a large scale, or perhaps they would not have born at all. The existence of visuo-motor links is supported by both behavioral and neural evidence, suggesting the interaction between the ventral and the dorsal streams. Early forms of reaching and grasping emerge around the fourth month of life, supporting that the dorsal stream would be already able to coordinate the motor output in response to the visual input mediated by the ventral stream (Braddick et al., 2003). Between the sixth and ninth month, children almost compulsorily reach and grasp any object within their arm's length (Newman et al., 2001), establishing and reinforcing rich perceptual-motor connections which will constitute scaffold for developing a broad visuo-motor neural architecture able to be activated even by less complex inputs (Pulvermüller, 1999; Martin et al., 2000). For example it has been shown that even just the observation of reaching movements activates the sensorimotor cortex in 14-month-old children with a stronger gradient as a function of older ages (Marshall et al., 2011). Similarly, visual perception of letters is associated with brain activations typical for the execution of handwriting movements (Longcamp et al., 2003), which in turn activate also visual regions typically involved in letter perception (James and Gauthier, 2006). Beyond action execution, vision can contribute also to accurate action planning, including the ability to anticipate the appropriate hand configuration to grasp a specific object (Rosenbaum et al., 1992). Typically, this ability is achieved at about 8 years (Smyth and Mason, 1997), but some delays can be encountered in presence of clinical conditions that are likely affect the interactions between the dorsal stream, ventral stream, and frontal brain areas (Braddick and Atkinson, 2013).

Not only can vision guide movements, but also motor training can affect visual perception. At the behavioral level, visuomotor training improves letter recognition in 5-year-old children (Bara and Bonneton-Botte, 2018). Similarly, handwriting improves after haptic (not visual) exploration of letters even at younger age (Bara et al., 2004), and is associated with better visual recognition of letters with respect to typing (Longcamp et al., 2005) and with better reading in general (Labat et al., 2010). At the neural level, in addition to the anatomo-functional overlap of brain regions activated by “seeing” and “doing” movements (Halje et al., 2015), already in 9-month-old children the motor components of the brain activity associated to observation of reaching actions occur earlier than the associated visual components (Southgate et al., 2009). This supports the existence of visuo-motor anticipation mechanisms based on experience-driven action understanding (Southgate et al., 2010). In addition to reaching and grasping, locomotion occupies an important position in visuo-motor coordination. In typically developing children locomotion emerges around the first year of life, strongly based on the ability of vision to provide information about the target position, possible obstacles, variations of surfaces, and edges. Thus, vision must have tight links also to the neural correlates of locomotion. Indeed already the simple observation of other children crawling or walking activates the sensorimotor cortex in 7–9 month-old children (De Klerk et al., 2015) as well as more frontal motor brain regions controlling locomotion in 14–16 month-old children (Van Elk et al., 2008), a resonance mechanism that persists in adulthood even just imagining to walk (Ionta et al., 2010).

## Visuo-Motor Neuropsychology

Conceiving bio-computational models to explain the causal link between dysfunctional neural networks and clinical phenotypes is the major challenge in neuropsychology. The following sessions offer an overview of the most common visual and visuo-motor disorders, including the possible associated neural explanations. Broadly, the following disorders have been classified as “lower” and “higher” level deficits, even if such a sharp distinction might not reflect all the details of each disorder. The “lower-level” classification comprises disorders mostly affecting the perceptual level, with a high importance of basic mechanisms associated with eye movements and convergence. The “higher-level” classification comprises disorders affecting levels beyond visual perception and rather extending to other spheres of human competences, such as motor and cognitive skills, eventually in absence of other possibly coherent deficits.

### Lower-Level Dysfunctions

#### Strabismus

Strabismus is one of the most common visual disorders, affecting the ability to maintain the alignment between the two eyes and therefore causing a binocularity breakdown due to the mismatch of the information provided by each eye to the visual cortex (Cullen, 2015). The importance of cortical mechanisms in the onset of strabismus is shown by the fact that, at least in monkeys, a lack of intervention at the cortical level can nullify the benefits brought by surgical treatment of the eye muscles (Pullela et al., 2018). Both in humans and other mammals, already the first weeks of life are fundamental for a proper oculomotor development leading to accurate eyes alignment (Tychsen, 2007). Due to the immaturity of V2 neurons with respect to V1 neurons, in the infant brain abnormal visual input can dramatically affect the neural wiring especially in V2 (Nakatsuka et al., 2007), the maturation of which could be misled by inappropriate experience/stimulation (Zheng et al., 2007). On this basis and in combination with the above-mentioned tight visuo-motor links, it is not surprising that the incongruent input received by V1 from the two eyes triggers a cascade of neural events ending in incongruent motor commands sent from to the oculomotor brain centers (e.g., the superior colliculus) back to the eyes (Das, 2016; Walton et al., 2017). Thus, the differential visual input of each eye would contribute to the misalignment of the eyes themselves, as supported by the inextricable relationship between sensory input and motor output (Perruchoud et al., 2014), including evidence that the onset of strabismus can derive from aberrant visual input (Chino et al., 1997). In addition to such aberrancies in the visual cortex, also disturbances in other brain areas have been linked to strabismus, such as abnormal visual-oculomotor behaviors in presence of dysfucntions in V5 and superior temporal gyrus (Mustari et al., 2008; Mustari and Ono, 2011), as well as other neuroanatomical aberrancies affecting the ventricles and corpus callosum (Ohtsuki et al., 2000). Such breakdowns in the visuo-motor loop can impair the perception of depth and also contribute to the onset of e.g., amblyopia (Sengpiel and Blakemore, 1996; Niechwiej-Szwedo et al., 2019).

#### Oculomotor Apraxia

Optic apraxia refers to the impossibility to perform eye movements, resulting in the so-called “sticky” vision: the impossibility to voluntarily shift gaze between different objects (Pena-Casanova et al., 1985). At the neural level, bilateral lesions in a fronto-parietal network comprising the frontal eye fields are considered at the origin of oculomotor apraxia, which therefore would not be necessarily associated strictly with dorsal stream damage (Leigh and Zee, 2015), extending to malformations/dysfunctions in the cerebellum (Shahwan et al., 2006) and midbrain (Jissendi-Tchofo et al., 2009; Merlini et al., 2010). In children, oculomotor apraxia can be present already around the 10th year of life (Tsao and Paulson, 2005), with a mean age of about 7 years and comprised between 2 and 18 years (Le Ber et al., 2003). Anatomo-functional aberrancies of the cerebellum have been repeatedly associated with oculomotor apraxia (Maria et al., 1999; Gleeson et al., 2004), with a particular responsibility for a too small cerebellar vermis (Sargent et al., 1997). The consequences of oculomotor apraxia do not remain limited to the visual domain, but rather spread on cognitive and social skills, especially in the case that oculomotor abilities are recovered too late (Kondo et al., 2007).

#### Amblyopia

Amblyopia can emerge when the visual input from one eye is not properly processed by the brain, which progressively develops a “preference” for the other eye. It results in atypical vision from one eye that otherwise appears organically normal (Bretas and Soriano, 2016). At the brain level, typical functional abnormalities associated with amblyopia converge in indicating V1 as the most affected brain region (Blakemore and Vital-Durand, 1986). However, the abnormal neural activity associated with amblyopia is not necessarily limited to V1, rather extending also to V2 and V3 (Barnes et al., 2001), even when V1 is normally functioning (Clavagnier et al., 2015). Interestingly, amblyopia patients present larger receptive fields in V1, V2, and V3, possibly as a consequence of the oculomotor instability of the amblyopic eye (Levin et al., 2010). Indeed there seem to be a sort of propagation of dysfunctional neural dynamics from V1 up to V5 (Barnes et al., 2001), which would result in specific deficits in extrastriate functions, including global motion (Simmers et al., 2003) or contrast-based contours (Wong et al., 2001).

#### Akinetopsia

Our ability to perceive motion allows us to distinguish objects from the background and to move in a three-dimensional world (Barton, 2011). Commonly called also motion blindness, akinetopsia refers to the impossibility to detect moving objects, in absence of scotoma (Zihl et al., 1983), while other low-level aspects like color or shape are normally detected (Zeki, 1991). Typically associated with an extrastriate brain lesion (Zihl et al., 1983; Cooper et al., 2012; Otsuka-Hirota et al., 2014), akinetopsia can indeed be experimentally induced by inhibiting V5 (Beckers and Hömberg, 1992), as well as V1 but at a smaller degree and with specific timing with respect to the visual stimulus (Beckers and Hömberg, 1992). This is in line with the observation that sensitivity to motion can survive cortical blindness (Ruffieux et al., 2016), also in children that present a congenital, but not acquired, lesion of V1 (Tinelli et al., 2013). While blindness to first-order motion (e.g., luminance-based) would result from lesions in V2/V3, blindness to second-order motion (e.g., contrast-based) would derive from lesions in V4/V5 (Cowey et al., 2006). A particular case of motion blindness is represented by the “form-from-motion” blindness, referring to the impossibility to detect forms on the basis of visual motion (Cowey and Vaina, 2000). Indeed form-from-motion blindness with and without akinetopsia are neurally dissociable, being the former associated with lesions in V5 and lateral occipital cortex and the latter with occipito-temporal regions (Blanke et al., 2007). Even if chronic cases have been reported (Cooper et al., 2012), akinetopsia seems a rather transient condition (Shipp et al., 1994), suggesting the existence of functionally neuroplastic changes able to establish alternative neural interactions to restore sensitivity to visual motion. Such a relative ease to naturally react to akinetopsia makes it difficult to detect, especially in populations characterized by high neural plasticity like children, where in fact akinetopsia is relatively rare and usually present in combination with the Alice in Wonderland syndrome as a results of encephalitis (Naarden et al., 2019).

### Higher-Level Dysfunctions

#### Optic Ataxia

Originally described by Rudolph Bálint in 1909 as part of a more complex syndrome (Rudolph Bálint, 1909), optic ataxia refers to the incapacity to perform accurate visually-guided movements, in absence of general motor impairments (Moreaud, 2003). Letting patients misplace the fork outside the plate, grasp a coffee mug from its body instead of its handle, or point to the wrong button on a computer keyboard, optic ataxia is considered the typical visuo-motor integration disorder (Teixeira et al., 2014). Not strictly limited to visuo-motor behaviors of the upper limb (Evans et al., 2013), it can emerge as early as in children aged between 5 (Dutton, 2003) and 10 years (Drummond and Dutton, 2007). Possibly as a consequence of premature birth (Dutton, 2013), optic ataxia has a confirmed association with aberrancies in the (occipital-parietal) dorsal stream (Philip et al., 2016). Indeed, the most accepted neural underpinnings of optic ataxia are comprised within the dorsal stream (Schindler et al., 2004), possibly also in interaction with the ventral stream (Himmelbach and Karnath, 2005). Further investigations reported that optic ataxia affects mainly the peripheral vision (Pisella et al., 2009) and is especially evident in contralesional visuo-motor tasks (Gaveau et al., 2008). This suggests that optic ataxia should not be considered as a unitary deficit, but rather presents various degrees and specifications as a function of the lesioned dorsal stream module responsible to coordinate visual perception and action. Nevertheless, recent evidence is starting to challenge such a sharp dissociation between perception and action in optic ataxia (Rossetti and Pisella, 2018). In particular, optic ataxia would derive from dorsal stream deficits in integrating multimodal sensory input (Jackson, 2010), it can be stimulus/task-specific (Hesse et al., 2014), and it can be bound to specific visuo-motor neurons located in different regions of the dorsal stream and beyond (Cooper and O'sullivan, 2016), especially the premotor cortex (Battaglia-Mayer and Caminiti, 2002) and a parietal-precuneus pathway (Teixeira et al., 2014).

#### Cerebral Visual Impairment

As one of the most common causes of visual impairment of cortical origin, cerebral visual impairment (CVI) can result from early brain damage, including a potentially large panel of correlated deficits beyond vision due to damages of the dorsal stream, the ventral stream, or both (Bennett et al., 2020). Behaviorally, it is possible to detect CVI by means of dedicated questionnaires (Gorrie et al., 2019; Fazzi and Micheletti, 2020). At the neural level, a relatively early detection of CVI is based on the analysis of visual evoked potentials which, already at 6 months of age, can appear abnormal and therefore suggest the presence of CVI (Mercuri et al., 1997b), further depending on the size (Mercuri et al., 1998) and location (Mercuri et al., 1997a) of the brain lesion. In particular, the basal ganglia seem to play a central role in coordinating the information exchanges between the eyes and the visual cortex, as well as in facilitating neural plasticity at the cortical level (Mikellidou et al., 2019), possibly resulting in aberrant patterns of anatomo-functional connectivity between different brain regions (Muñoz-Moreno et al., 2016; Bathelt et al., 2020). It is anyways important to note that CVI can impair a full range of competences at different levels, including purely visual skills (visual field, motion sensitivity, visual exploration) as well as attention, memory, and visuo-motor coordination (Lueck et al., 2019). This is the main reason why current trends in neuro-ophthalmology highlight the importance of considering each patient as an individual case that should be evaluated on the basis of a personalized and multidisciplinary assessment combining ophthalmology, neuropsychology, and pedagogy (Ortibus et al., 2019).

#### Dorsal Stream Vulnerability

As already outlined, the dorsal stream is considered the main neural architecture processing spatial aspects of vision and their translation into relevant information for functions beyond the mere sight. Converging evidence supports that the dorsal stream is more vulnerable than the ventral stream to developmental disorders (Grinter et al., 2010), due to genetic or contextual factors (Atkinson, 2017) as well as interventional approaches (Tonks et al., 2019). Possibly leading to cognitive decline (Ricci et al., 2015), attentional/visuo-spatial deficits (Tonks et al., 2019), and visuo-motor impairments (Atkinson and Braddick, 2011), the dorsal stream vulnerability can start in early age and keeps affecting the individual competences from early childhood across the life span (Sciberras-Lim and Lambert, 2017). Nevertheless, recent findings are starting to challenge this view, by arguing that dorsal stream vulnerability might be stimulus-specific rather than a general dysfunction (Joshi et al., 2020), as shown for example by the relatively preserved motion sensitivity in amblyopia (Hamm et al., 2014). Beyond the stimulus-specificity, such a controversy might result also from task-specificity since, for example, some dorsal stream functions (e.g., time estimation and attentional tasks) seem more sensitive to developmental disorders than others (e.g., numerical discrimination or mapping). In consideration of such a variability among stimuli and tasks, it is clear that to evaluate a wide range of symptoms like those related to dorsal stream vulnerability implies the need of using multidimensional scales for evaluating dorsal stream vulnerability (Atkinson et al., 2002).

#### Developmental Coordination Disorder

The diagnosis of Developmental Coordination Disorder (DCD) is based on the presence of motor impairments in absence of other neuropsychological deficits able to explain patient's poor motor performance (cerebral palsy, neurodegeneration, traumatic brain injuries, etc.) (Blank et al., 2019). The characteristics of DCD include impaired control of ocular, postural, and manual tasks, as well as motor imagery (Adams et al., 2014). One of the possible interpretations of DCD explains the disorder as the result of breakdowns in a visuo-motor matching system which would allow to perform movements on the basis of observing the same movements performed by somebody else (Werner et al., 2012). Such a breakdown would affect in particular the ability to process and exploit the temporal binding between vision and movements (Nobusako et al., 2018). The visuo-motor interpretation of DCD is in line with evidence that DCD patients exhibit impaired visuo-motor skills (Reynolds et al., 2017) and decreased brain activation in regions typically involved in visuo-motor imitation (Licari et al., 2015) and action planning (Reynolds et al., 2019). In particular, even if a large consensus has not been reached yet (Brown-Lum and Zwicker, 2015), it seems that the brain dysfunctions associated with DCD are mainly located in associative regions of the parietal and frontal lobe particularly important for visually-based action imitation (Biotteau et al., 2016). In sum, despite the little number of studies and the large variability of their results, the is a tendency to consider DCD as a visuo-motor integration deficit specifically affecting the neural network responsible for visually interpreting actions performed by other people and exploit such information for guiding self-produced movements. However, further studies are required and the present conclusions have to be regarded with caution.

#### Prosopagnosia

The ability to recognize faces is one of the most important abilities in the human world. The centrality of this function is reflected in the fact that the brain dedicates a specific neural substrate to process face-like visual input (Zeugin et al., 2020), with a particular emphasis on the fusiform face area in the (ventral stream) inferior temporal cortex (Kanwisher et al., 1997). Prosopagnosia refers to the inability to recognize faces (Mayer and Rossion, 2007) associated with occipito-temporal brain activity (Dalrymple et al., 2014) and, in particular, with bilateral lesion of the fusiform face area (Grüter et al., 2008). It is dissociated from other objet-recognition deficits as, for example, there are cases in which in consequence of a bilateral fusiform lesion patients become unable to recognize faces while their performance in object recognition remains at good levels (Moscovitch et al., 1997). Since prosopagnosic people are largely unaware of their deficit, prosopagnosia can dramatically affect the cognitive development of otherwise typically growing children (Schmalzl et al., 2008), including the preference for social identification on the basis of whole-body configuration instead of facial features (Wilson et al., 2010). This conditions can trigger a cascade of aversive events and behaviors also in daily contexts like schools, where both teachers and colleagues would not detect the prosopagnosic deficit and therefore might put disproportionate reactions in place (Wilson et al., 2010). To prevent and possibly overcome this risk, at present there are strong trends toward the development of specific test to assess face perception abilities in children, such as the Dartmouth Database of Children's Faces (Dalrymple et al., 2013) and the Cambridge Face Memory Test for Children (Croydon et al., 2014).

#### Somatoparaphrenia

The ability to recognize our own body also plays a central role in adaptive behaviors and consciousness (Ionta et al., 2013). Somatoparaphrenia refers to the inability to identify one's own body part as belonging to one self, both at the subjective conscious (Invernizzi et al., 2013) and objective physiological levels (Romano et al., 2014). Despite its psychiatric component (Feinberg and Venneri, 2014), somatoparaphrenia is largely associated with unilateral lesions, mostly in the right hemisphere and therefore affecting the left side of the body (Vallar and Ronchi, 2009). At a more specific neural level the available evidence is controversial, with clinical observations reporting damages in either the dorsal or the ventral stream, as well as other brain regions. Thus, different studies proposed that somatoparaphrenia would derive from lesions in the posterior insula (Baier and Karnath, 2008), supramarginal gyrus (Feinberg et al., 1990), orbito-frontal regions (Feinberg et al., 2010), posterior superior temporal cortex (Vallar and Ronchi, 2009). In addition, recent investigations highlighted the importance of more complex fronto-temporal-parietal cortical networks as well as subcortical circuits (Gandola et al., 2012). Even if somatoparaphrenia is commonly associated with hemispatial neglect, it can be present also in isolation and associated with specific subcortical lesions, comprising the basal ganglia, thalamus, and internal capsule (Invernizzi et al., 2013). Interestingly when somatoparaphrenic patients observe the misrecognized body part in a mirror (as from a third-person perspective), their self-misattribution decreases (Fotopoulou et al., 2011). Already rarely detected in adults, possibly due to its comorbidity with hemispatial neglect and its confusion with asomatognosia, evidence of somatoparaphrenia in children is even more scarce. However, a study implementing a neuroinvestigation technique with high spatial resolution (electrocorticography) in awake humans, reported that following abnormal neural firing in the right occipito-temporo-parietal cortex, a 10-year-old child reported somatoparaphrenic symptoms, being unable to recognize his left hand (Heydrich et al., 2011). Altogether, it seems that the small numbers to somatoparaphrenic reporting reflects a general lack of episodes spontaneously mentioned by the patients together with the confusion with other pathologies by the evaluators. For this reasons, it would be important to explicitly assess somatoparaphrenic symptoms using structured interviews (Brandt et al., 2005) and/or standardized scales especially in developing age.

#### Hemispatial Neglect

The absence of perception and action in half of the sensory fields and peri-personal space, respectively, defines the hemispatial neglect. Patients suffering from this syndrome to not perceive sensory stimuli in any modality from the neglected hemifield and do not perform movements in that hemi-peri-personal space. The traditional test to assess neglect is the line bisection task, in which patients are presented with a paper sheet with a number of short lines distributed all over a paper sheet. Typically, when patients are asked to draw a line over each short line (bisection), they mark only half of the lines (those located in the non-neglected hemifield). At the neural level, hemispatial neglect seems to derive from dysfunctions in the right inferior parietal lobule, possibly in association with deteriorated input from the ventral stream (Milner and Goodale, 2008) or with impaired ventro-fronto-parietal circuit distinct from the traditional dorsal stream (Husain and Nachev, 2007). However, hemispatial neglect can emerge also following lesions of the frontal cortex (Husain and Kennard, 1996), basal ganglia or thalamus (Mort et al., 2003), as well as from lesions of white matter pathways connecting the parietal and frontal cortex (Bartolomeo et al., 2007). Altogether, it seems that hemispatial neglect may be the result of a lesions in a large-scale cortico-subcortical network, possibly implicated in attention-related abilities. Interestingly, while most intervention protocols eventually produce only temporary improvements, the most efficient and relatively long-lasting treatment is based on the use of prism adaptation (Rossetti et al., 1998). In particular, the visual distortion brought by wearing prism lenses would trigger the activation of otherwise silent visuo-motor circuits as valid alternative neural pathways to allow visuo-motor coordination, with benefit spreading also in the cognitive domain (Rossetti et al., 1998). Even if hemispatial neglect is commonly associated with adult and elderly patients, also children can be affected, and not necessarily only in the visual domain (Martin and Trauner, 2019). Cases of hemispatial neglect have been reported for children as young as 3-year-old (Thompson et al., 1991), 6-year-old (Ferro et al., 1984), and above (Hausmann et al., 2003; Marsh et al., 2009). Actually, also at 6 months after birth, children with pre- or post-natal brain damage can exhibit otherwise unmotivated preference for interacting with objects located in the hemi-peri-personal space ipsilateral to a unilateral lesion in the left or right hemisphere (Trauner, 2003). Most of the studies indicate that children can relatively quickly recover from neglect symptoms within a few weeks (Kleinman et al., 2010) or months (Thompson et al., 1991) after a stroke. Even children that suffered from a perinatal stroke, especially in the right hemisphere, can present hemi-neglect-like symptoms in the left hemi-field and peri-personal space, including visuo-motor deficits (Vicari et al., 1998), reaching and grasping (Trauner, 2003), as well as visual cancellation and manual exploration (Thareja et al., 2012). These studies further showed that, in contrast with the typical right-hemispheric dominance of hemi-spatial neglect in adults, in children a more dramatic bilateral neglect can result from a left-hemispheric lesion (Trauner, 2003; Thareja et al., 2012), whose resolution might require maturation up to adolescence or adulthood (Yousefian et al., 2015).

## Final Remarks

Understanding the behavioral and neural fundaments of the complex interaction between vision and other sphere of human life is the prerequisite for better targeted interventional procedures in case of deficits, as well as for more efficient training programs in typically developing populations. As a very general overview, the present paper summarizes some of the most relevant evidence about the neural basis of vision and associated abilities in development and beyond. With the aim of constituting a first-glance reference for researchers and clinicians interested in vision and visuo-motor integration, this review hopes to guide and trigger further investigations toward more specific publications in case of specific interests.

Establishing the neural correlates of aberrant behaviors helps identifying the neural networks responsible for a given function which, in turn, can boost the development of more effective training and rehabilitation protocols. Accordingly, the knowledge summarized here sustains the importance of adopting a systemic approach even in the evaluation of the impact of supposedly purely visual deficits, which indeed can affect also motor skills, cognition, social skills, and emotional processing. Addressing such a complexity is the fundamental requirement of current implementations of systemic approaches for visually-related training in typical conditions or in response to visual disorders, including virtual reality (Adams et al., 2018; Choi et al., 2021), robotics (Mirkowski et al., 2019; Zhexenova et al., 2020), and touch screen technology (Aslam et al., 2016; Sheehan and Uttal, 2016; Dalecki et al., 2019).

## Author Contributions

SI conceived the work, performed the literature analysis, and wrote the manuscript.

## Conflict of Interest

The author declares that the research was conducted in the absence of any commercial or financial relationships that could be construed as a potential conflict of interest.
